# Risk factor analysis and nomogram for predicting in-hospital mortality in ICU patients with sepsis and lung infection

**DOI:** 10.1186/s12890-021-01809-8

**Published:** 2022-01-07

**Authors:** Yinlong Ren, Luming Zhang, Fengshuo Xu, Didi Han, Shuai Zheng, Feng Zhang, Longzhu Li, Zichen Wang, Jun Lyu, Haiyan Yin

**Affiliations:** 1 Intensive Care Unit, The First Affiliated Hospital of Jinan University, Guangzhou, 510630 Guangdong Province People’s Republic of China; 2 Department of Clinical Research, The First Affiliated Hospital of Jinan University, Guangzhou, 510630 Guangdong Province People’s Republic of China; 3 School of Public Health, Xi’an Jiaotong University Health Science Center, Xi’an, Shaanxi Province People’s Republic of China; 4 School of Public Health, Shaanxi University of Chinese Medicine, Xianyang, Shaanxi Province People’s Republic of China; 5 Department of Public Health, University of California, Irvine, Irvine, CA USA

**Keywords:** Sepsis, Nomogram, Lung infection

## Abstract

**Background:**

Lung infection is a common cause of sepsis, and patients with sepsis and lung infection are more ill and have a higher mortality rate than sepsis patients without lung infection. We constructed a nomogram prediction model to accurately evaluate the prognosis of and provide treatment advice for patients with sepsis and lung infection.

**Methods:**

Data were retrospectively extracted from the Medical Information Mart for Intensive Care (MIMIC-III) open-source clinical database. The definition of Sepsis 3.0 [10] was used, which includes patients with life-threatening organ dysfunction caused by an uncontrolled host response to infection, and SOFA score ≥ 2. The nomogram prediction model was constructed from the training set using logistic regression analysis, and was then internally validated and underwent sensitivity analysis.

**Results:**

The risk factors of age, lactate, temperature, oxygenation index, BUN, lactate, Glasgow Coma Score (GCS), liver disease, cancer, organ transplantation, Troponin T(TnT), neutrophil-to-lymphocyte ratio (NLR), and CRRT, MV, and vasopressor use were included in the nomogram. We compared our nomogram with the Sequential Organ Failure Assessment (SOFA) score and Simplified Acute Physiology Score II (SAPSII), the nomogram had better discrimination ability, with areas under the receiver operating characteristic curve (AUROC) of 0.743 (95% C.I.: 0.713–0.773) and 0.746 (95% C.I.: 0.699–0.790) in the training and validation sets, respectively. The calibration plot indicated that the nomogram was adequate for predicting the in-hospital mortality risk in both sets. The decision-curve analysis (DCA) of the nomogram revealed that it provided net benefits for clinical use over using the SOFA score and SAPSII in both sets.

**Conclusion:**

Our new nomogram is a convenient tool for accurate predictions of in-hospital mortality among ICU patients with sepsis and lung infection. Treatment strategies that improve the factors considered relevant in the model could increase in-hospital survival for these ICU patients.

**Supplementary Information:**

The online version contains supplementary material available at 10.1186/s12890-021-01809-8.

## Background

Sepsis is particularly common in ICUs, and it is one of the main causes of disability and death in severely ill patients. At least 3 million patients worldwide suffer from sepsis annually, with mortality rates as high as 30–50%, causing serious damage to both their families and society as a whole [1–3]. Sepsis usually has rapid onset, rapidly deteriorating progress, affects multiple organs, and is difficult to reverse, meaning that when a patient is diagnosed with sepsis, it should be treated immediately.

Sepsis has a complicated source and dangerous condition. It can be caused by various factors and affects the function of multiple organs. Lung infection is one of the main causes of sepsis [4, 5], and more than 40% of sepsis patients have lung infections. Studies have indicated that patients with sepsis and lung infection are more ill and have a higher mortality rate [6, 7]. If patients with sepsis and lung infection can be assessed early, and appropriate treatment strategies can be applied in time, mortality rates can be effectively reduced [8]. However, there is no effective scale for evaluating the prognosis and condition of sepsis patients with lung infection, leading to precise treatment delay and extra plague for patients. Although the Sequential Organ Failure Assessment (SOFA) score and Acute Physiology and Chronic Health Evaluation II (APACHEII) scores are somewhat useful in assessing the condition of patients, the scores mainly evaluate the physiological functions of organs and lack pertinence and sensitivity in evaluating clinical practices and prognoses of patients with sepsis and lung infection and are not effective in guiding the treatment of these patients during the clinical process. As there is currently no effective evaluation scale for predicting the in-hospital outcomes of patients with sepsis and lung infection, our study analyzed the risk factors of in-hospital mortality for sepsis patients with lung infection from the Medical Information Mart for Intensive Care (MIMIC-III) database, constructed a nomogram prediction model, and compared it with the SOFA score and Simplified Acute Physiology Score II (SAPSII) systems to accurately evaluate patient conditions, predict the prognostic outcome, and provide advice for the treatment of patients with sepsis and lung infection.

## Data and method

### Database

Research data were extracted from the MIMIC-III database, which includes ICU patients who visited the Beth Israel Deaconess Medical Center [8, 9]. Structured Query Language (SQL) with Navicat Premium was used to search for and extract data, and R software was used for further processing of the data. The MIMIC-III database (version 1.4, https://mimic.physionet.org/) is free for public use and contains information required for clinical research, such as basic demographic characteristics, examination results, disease diagnoses, and treatment methods received. The in-hospital and postdischarge outcomes of patients are also included in the database. After obtaining access to the MIMIC-III database and receiving approval from the institutional review boards of both Beth Israel Deaconess Medical Center (Boston, MA, USA) and the Massachusetts Institute of Technology (Cambridge, MA, USA), we were free to extract data for our study. Since the identity and private messages of all patients has been absolutely concealed within the database, our study does not violate the privacy of the patients.

### Patient admission and data extraction

The following inclusion criteria were applied: (1) entering the ICU for the first time, (2) diagnosed with sepsis and lung infection, and (3) aged 18–80 years. The exclusion criteria were (1) SOFA score < 2 and (2) stay of shorter than 24 h in the ICU.

The definition of Sepsis 3.0 [10] was used, which includes patients with life-threatening organ dysfunction caused by an uncontrolled host response to infection, and SOFA score ≥ 2. Lung infection diagnosis was determined by the ninth edition of the International Classification of Diseases codes for the MIMIC-III patient data.

**Fig. 1:**
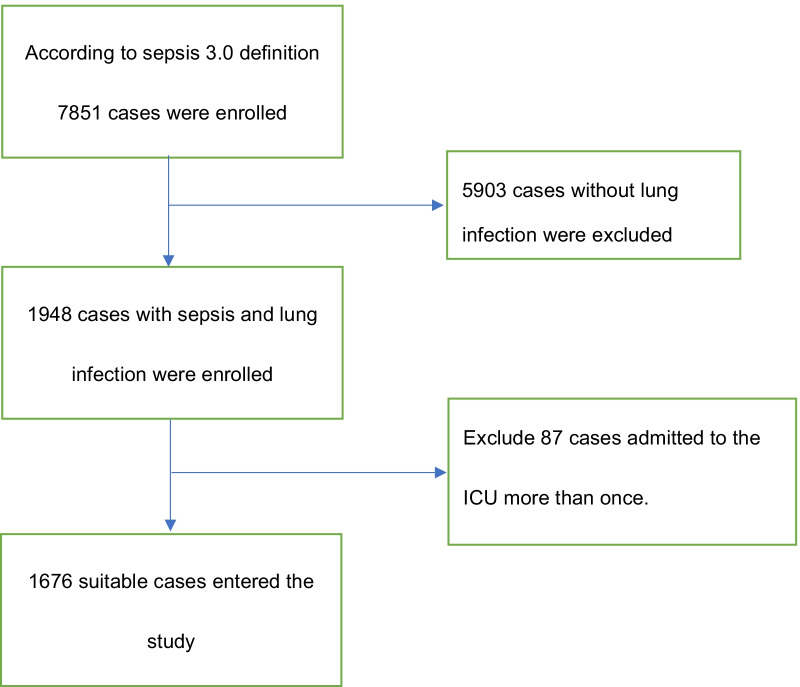
Flow chart of patient selection (MIMIC-III, Medical Information Mart for Intensive Care I.)

Data were extracted from the MIMIC-III database using SQL with Navicat Premium. The sampling process is presented in Fig. 1. We extracted patients diagnosed sepsis and pneumonia(including all pathogen infection like bacteria, virus and fungus) according to the ICD-9,and then extracted ID, SOFA score, and basic characteristics of target patients from MIMIC-III data tables, and unsuitable patients (< 18 or ≥ 80 years old, SOFA score < 2, and ICU stay of < 24 h) were excluded. The extracted variable items were (1) baseline characteristics and vital signs including mean arterial pressure (MAP), heart rate, respiratory rate, SpO2, and temperature, (2) laboratory test and blood culture results, (3) SOFA score, SAPSII, and GCS to assess organ function, (4) complications including congestive heart failure(CHF), chronic obstructive pulmonary disease(COPD), renal failure, liver disease, neurological disease, cancer, diabetes, AIDS, organ transplantation, and pneumomycosis, and (5) the use of vasopressor and interventions such as CVP, CRRT, MV, and fiberscopy. Vital signs and laboratory data from blood examinations, basic blood biochemical indexes, and arterial blood gas were collected over the first 24 h of the ICU stay. If a variable was recorded more than once, the value representing the most severe illness was used. We collected discharge outcome data of all patients as the dependent variable of this observational study.

### Data management and statistical analysis

We used multiple imputation method to fill in missing data through R software package of “lattice”, “ MASS”, “nnet” and “mice”, variables with a missing data rate of more than 20% were deleted [11]. After confirming the data set, the continuous variables of blood glucose, MAP, pH, heart rate, and body temperature were converted into graded categorical variables (the concrete transformations are presented in Table 1), mean ± standard deviation or median (interquartile range) values described continuous variables, and categorical variables were presented as percentages.

**Table 1:** Demographic and clinical characteristics of patients

	Survived to discharge N = 1191		Died in hospital N = 485	*P* value
**Demographics**				
Age (years)		58.84 ± 13.95	62.13 ± 12.60	< 0.001*
Gender (%)				
Male		724 (60.8)	283 (58.4)	0.385
**Vital signs**				
Heart Rate (times/min, %)	0 (60–100)	358 (30.1)	127 (26.2)	
	1 (101–120)	418 (35.1)	146 (30.1)	0.003*
	2 (< 60or 121–140)	276 (23.2)	134 (27.6)	
	3 (140–160)	118 ( 9.9)	58 (12.0)	
	4 (> 160)	21 (1.8)	20 (4.1)	
MBP (mmhg, %)	0 (65–110)	226 (19.0)	81 (16.7)	0.191
	1 (50–64 or 111–130)	618 (51.9)	242 (49.9)	
	2 (< 50 or > 160)	347 (29.1)	162 (33.4)	
Breath rate (times/min, %)	0 (12–24)	254 (21.3)	96 (19.8)	0.525
	1 (10–11 or 25–34)	641 (53.8)	252 (52.0)	
	2 (35–49)	272 (22.8)	127 (26.2)	
	3 (< 6 or > 49)	24 (2.0)	10 (2.1)	
T (℃, %)	0 (36–37.3)	333 (28.0)	184 (37.9)	< 0.001*
	1 (37.3–38.2 or 35.001–35.9)	399 (33.5)	177 (36.5)	
	2 (38.201–39.1or 34.001–35)	299 (25.1)	81 (16.7)	
	3 (39.101–40 or < 34)	135 (11.3)	32 (6.6)	
	4(> 40)	25 (2.1)	11 (2.3)	
SpO2		92.00 [89.00, 95.00]	91.00 [88.00, 94.00]	< 0.001*
Gcs		15.00 [14.00, 15.00]	15.00 [12.00, 15.00]	0.002*
Sofa		6.00 [4.00, 9.00]	8.00 [5.00, 11.00]	< 0.001*
SAPSII		40.00 [31.00, 49.00]	49.00 [41.00, 59.00]	< 0.001*
**Library tests**				
Glucose (mmol/L, %)	0 (3.9–7.8)	380 (31.9)	138 (28.5)	0.5
	1 (7.8–11.1)	390 (32.7)	167 (34.4)	
	2 (11.2–16.7 or 1.6–3.8)	276 (23.2)	123 (25.4)	
	3 (> 16.7 or < 1.6)	145 (12.2)	57 (11.8)	
ALBUMIN (g/dl)		2.82 ± 0.66	2.66 ± 0.68	< 0.001*
BILIRUBIN (mg/dl)		0.70 [0.40, 1.30]	0.90 [0.50, 2.70]	< 0.001*
BUN (mg/dl)		29.00 [18.00, 47.00]	37.00 [24.00, 58.00]	< 0.001*
CREATININE (mg/dl)		1.40 [0.90, 2.40]	1.60 [1.10, 2.80]	< 0.001*
WBC (k/ul)		13.80 [9.60, 19.90]	14.50 [8.30, 20.90]	0.836
HEMOGLOBIN (g/dl)		9.62 ± 1.90	9.32 ± 1.90	0.001*
PLATELET (k/ul)		180.00 [112.00, 264.00]	144.00 [60.00, 247.00]	< 0.001*
APTT(s)		34.50 [29.45, 45.70]	37.80 [30.80, 55.00]	< 0.001*
INR		1.40 [1.20, 1.80]	1.60 [1.30, 2.40]	< 0.001*
PT(s)		15.20 [13.70, 18.00]	16.40 [14.20, 21.20]	< 0.001*
TnT (ug/L)		0.08 [0.03, 0.27]	0.10 [0.04, 0.37]	< 0.001*
NLR		8.57 [6.24, 9.94]	9.38 [7.36, 35.52]	< 0.001*
Blood culture (%)		413 (34.7)	193 (39.8)	0.055
**Blood gas analysis**				
PH (%)	0 (7.35–7.45)	426 (35.8)	139 (28.7)	0.011*
	1 (7.25–7.34 or 7.46–7.55)	434 (36.4)	175 (36.1)	
	2 (7.15–7.24 or 7.56–7.65)	220 (18.5)	105 (21.6)	
	3 (7.05–7.14)	82 ( 6.9)	47 ( 9.7)	
	4 (< 7.05or > 7.66)	29 ( 2.4)	19 ( 3.9)	
PO2 (mmhg)		87.35 ± 45.86	84.21 ± 45.24	0.042
PCO2 (mmhg)		48.13 ± 16.00	49.58 ± 17.67	0.243
Oxygenation index		160.00 [104.64, 237.00]	147.00 [95.00, 224.00]	0.017*
BICARBONATE (mmol/L)		21.00 [18.00, 24.00]	20.00 [17.00, 23.00]	0.002*
LACTATE (mmol/L)		2.10 [1.40, 3.65]	2.70 [1.70, 4.80]	< 0.001*
**Complication**				
CHF (%)		427 (35.9)	188 (38.8)	0.287
COPD (%)		332 (27.9)	154 (31.8)	0.127
Renal failure (%)		240 (20.2)	129 (26.6)	0.005*
Liver disease (%)		209 (17.5)	162 (33.4)	< 0.001*
Neurologic disease (%)		184 (15.4)	65 (13.4)	0.321
Cancer (%)		112 ( 9.4)	99 (20.4)	< 0.001*
Diabetes (%)		390 (32.7)	143 (29.5)	0.214
Aids (%)		14 ( 1.2)	7 ( 1.4)	0.838
Organ transplanted (%)		79 ( 6.6)	57 (11.8)	0.001*
Pneumomycosis (%)		26 ( 2.2)	21 ( 4.3)	0.024*
**Intervention**				
Vasopressors (%)		732 (61.5)	362 (74.6)	< 0.001*
Vasopressor maximum dose [ug/(dl•min)]		1.00 [0.00, 3.00]	2.00 [0.00, 5.00]	< 0.001*
MV (%)		850 (71.4)	406 (83.7)	< 0.001*
CRRT (%)		168 (14.1)	136 (28.0)	< 0.001*
CVP (%)		658 (55.2)	290 (59.8)	0.099
Max CVP value (mmhg)		9.00 [9.00, 20.00]	9.00 [9.00, 20.00]	0.509
Fiberscope (%)		71 ( 6.0)	37 ( 7.6)	0.25

Heart rate, mean arterial pressure, body temperature, PH value, and Glucose was converted into graded categorical variables

Continuous data are presented as mean ± standard deviation (SD), and median (interquartile ranges),categorical data are presented as frequency (percentage)

CHF congestive heart failure; COPD chronic obstructive pulmonary disease; MBP mean blood pressure; NLR neutrophils to lymphocytes ratio; MV mechanical ventilation; CRRT continuous renal replacement treatment; CVP central vein pressure

We then conducted logistic regression analysis in training set to determine independent variables relating to patient in-hospital mortality, selected related variables by using multivariate logistic regression analysis on the independent variables to calculate estimated odds ratios (ORs) and 95% confidence intervals (CIs), and set *p* < 0.1 as the threshold for excluding nonsignificant factors. The development of the nomogram began with randomly dividing samples without replacement into a training set and a validation set at a ratio of 7:3. An original nomogram for predicting in-hospital mortality of patients with sepsis and lung infection was constructed using the training set. Parsimony was a goal in the modeling, the most effective model should achieve the study aim and contain as few variables as possible [12], and considering clinical practice, GCS score and oxygenation index were included in the nomogram to complete the optimized nomogram. Areas under the receiver operating characteristic curves (AUROCs) were calculated to evaluate the performance of the nomogram [13], and we compared the AUROC of nomogram with those of SOFA score and SAPSII in both the training and validation set through Delong’s test. The integrated discrimination improvement (IDI) and net reclassification index (NRI) were used to compare discrimination slopes. The Hosmer–Lemeshow test and calibration plots were used to evaluate the calibration of the model [14]. Finally, to evaluate the net benefits, decision-curve analysis (DCA) was applied to the nomogram, SOFA score, and SAPSII models, and then these were compared under different threshold probabilities in the training and validation sets.

R software (version 3.6.1, R Foundation for Statistical Computing, Vienna, Austria) was used for all statistical analyses. To amend incomplete data, the multiple imputation method was applied during the statistical analysis process. The statistically significant threshold was a two-sided p value of < 0.05. TRIPOD (transparent reporting of a multivariate prediction model for individual prognosis or diagnosis) checklist for our model development and validation is in the Additional file 1 according to the guidelines [15].

## Results

### Participant characteristics

After the inclusion and exclusion criteria were applied to the data set, 1676 patients with sepsis and lung infection were included in our study (Fig. 1). The demographic and clinical characteristics of all participants are listed in Table 1; age, heart rate, body temperature, SpO2, GCS score, SOFA score, SAPSII, albumin, and creatinine differed significantly between the death and survival groups.

### Logistic regression variable screening results and nomogram development

**Fig. 2:**
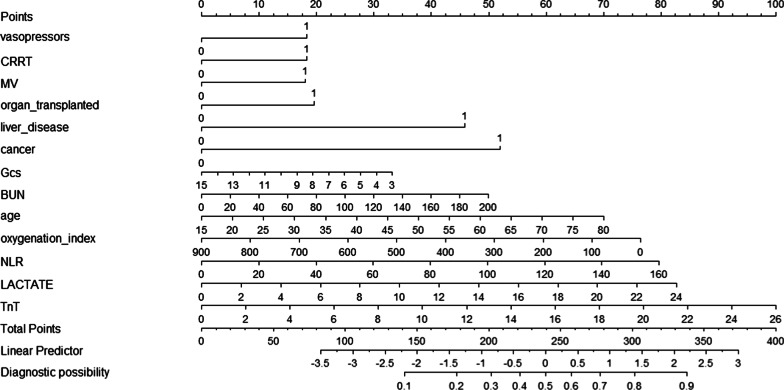
Nomogram for predicting in-hospital-mortality of patients with sepsis and lung infection. When using it, drawing a vertical line from each variables upward to the points and then recording the corresponding points (i.e., “age = 80” = 70 points). The point of each variable was then summed up to obtain a total score that corresponds to a predicted probability of in-hospital-mortality at the bottom of the nomogram

The risk factors relating to in-hospital mortality of patients with sepsis and lung infection after multivariate logistic regression are listed in Table 2. AIC of deleted variables in stepwise logistic regression are shown in Table 3. Since the GCS score is an important indicator of the conscious state, and oxygenation index is a more accurate factor than SpO2 in reflecting oxygenation function in clinical practice, a model was established with the variables of temperature, oxygenation index, age, BUN, lactate, GCS score, liver disease, cancer, organ transplantation, TnT, neutrophil-to-lymphocyte ratio (NLR), and CRRT, MV and vasopressor use. The nomogram shown in Fig. 2 for predicting in-hospital mortality of patients with sepsis and lung infection was constructed based on this model.

**Table 2:** Risk factors related to hospital deaths of patients identified by multivariable logistic regression

Variables	OR	95% CI	*P* value
Age	1.02	1.01–1.03	0.048
SAPSII	1.05	1.04–1.06	< 0.001
Heart Rate	1.19	1.08–1.31	0.060
MBP	1.07	0.99–1.16	0.090
Body temperature	0.77	0.69–0.86	0.009
SpO2	0.97	0.96–0.98	0.001
BICARBONATE	0.98	0.96–1	0.041
BUN	1.01	1.00–1.01	0.005
CREATININE	1.02	0.97–1.07	< 0.001
LACTATE	1.12	1.08–1.17	< 0.001
INR	1.16	1.08–1.24	0.082
Renal failure	1.44	1.12–1.84	0.01
Liver disease	2.36	1.85–3	< 0.001
Cancer	2.47	1.84–3.32	< 0.001
Organ transplanted	1.87	1.31–2.68	0.017
Vasopressors	1.85	1.46–2.34	0.090
MV	2.06	1.57–2.71	0.077
CRRT	2.37	1.84–3.07	< 0.001
TnT	1.1	1.03–1.18	0.009
NLR	1.0143	1.01–1.02	< 0.001
Blood culture	1.25	1–1.55	0.041

**Table 3:** AIC of deleted variables in stepwise logistic regression

Deleted variable	AIC
Fiberscope	−2.00
Gender	−4.00
PH	−11.34
APTT	−13.33
HEMOGLOBIN	−15.29
WBC	−20.87
Max CVP value	−22.77
Vasopressor maximum Dose	−24.67
PO2	−26.52
Pneumomycosis	−28.31
PT	−30.08
Glucose	−34.41
Breath rate	−38.62
CHF	−40.44
Neurologic disease	−42.17
Aids	−43.46
Diabetes	−44.53
CVP	−46.31
Oxygenation index	−45.83
PCO2	−46.21
Blood culture	−46.20
PLATELET	−42.01
COPD	−40.17
ALBUMIN	−38.19
TnT	−35.64
BILIRUBIN	−33.06

### Performance of the nomogram model

**Fig. 3:**
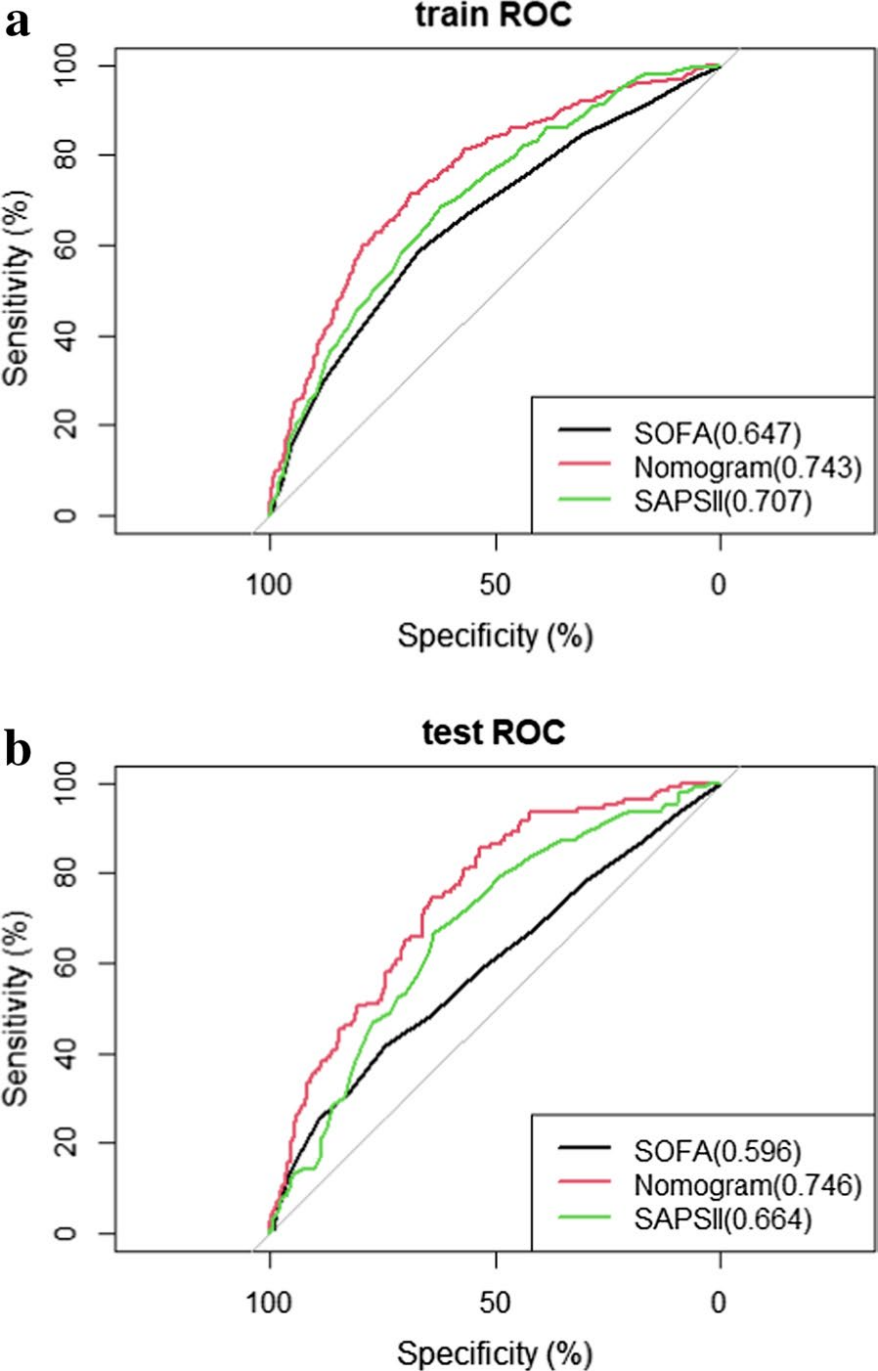
ROC curve and AUROC of SOFA, nomogram and SAPSII in training set (**a**) and validation set (**b**).The AUROC of nomogram is bigger than it of SOFA and SAPSII in both training set and validation set

We first used AUROC (Fig. 3, Table 4) to evaluate the effect of nomograph and found in training set our nomogram’ AUROC is higher than SOFA (0.743 vs 0.647) and SAPSII (0.743 vs 0.707); and in validation set nomogram’ AUROC is higher than SOFA (0.746 vs 0.596) and SAPSII (0.746 vs 0.664), indicating that the nomogram is more effective in predicting in-hospital mortality of patients in our study. Delong’s test was used for testing the difference of AUROC between SOFA, SAPS II scores and the nomogram model (Table 4). In training set, result of AUROC between SOFA and the nomogram is Z = −5.0879 (*p* < 0.01), result of AUROC between SAPII and the nomogram is Z = 2.8677 (*p* = 0.004); in validation set, result of AUROC between SOFA and the nomogram is Z = −5.5984 (*p* < 0.01), result of AUROC between SAPII and the nomogram is Z = 2.7171 (*p* = 0.007). Our model had significantly better predictive accuracy than SAPSII and SOFA score in both the training and validation sets.

**Table 4:** The AUROC and IDI of SOFA, SAPSII and Nomogram in training set and validation set

Predictive Model	AUROC		*P*^a^ value	IDI	*P* value
Training set	Nomogram	0.743 (0.713–0.773)			
	SOFA	0.647 (0.616–0.684)	< 0.001	9.73% (7.48–11.98%)	< 0.001
	SAPSII	0.707 (0.668–0.741)	0.004	6.84% (4.60–9.08%)	< 0.001
Validation set	Nomogram	0.746 (0.699–0.790)			
	SOFA	0.596 (0.543–0.654)	< 0.001	14.06% (10.71–17.41%)	< 0.001
	SAPSII	0.664 (0.613–0.715)	0.007	11.68% (8.06–15.30%)	< 0.001

AUROC area under the receiver operating characteristic curve; IDI integrated discrimination improvement

^a^Delong’s test was used for testing the difference of AUROC between SOFA SAPS II scores and the nomogram model. In training set, result of AUROC between SOFA and the nomogram is Z = −5.0879 (*p* < 0.01), result of AUROC between SAPII and the nomogram is Z = 2.8677 (*p* = 0.004); in validation set,result of AUROC between SOFA and the nomogram is Z = −5.5984 (*p* < 0.01), result of AUROC between SAPII and the nomogram is Z = 2.7171 (*p* = 0.007)

The NRIs of the nomogram were 47.32% (95% C.I.:36.08–70.46%) and 36.42% (95% C.I.:16.11–59.11%) in the training and validation sets, respectively**.** Moreover, the IDI (Table 4) of the nomogram was significantly higher than that of the SOFA score and SAPSII in both sets, indicating that the nomogram has better discrimination performance than the SOFA score and SAPSII models.

**Fig. 4:**
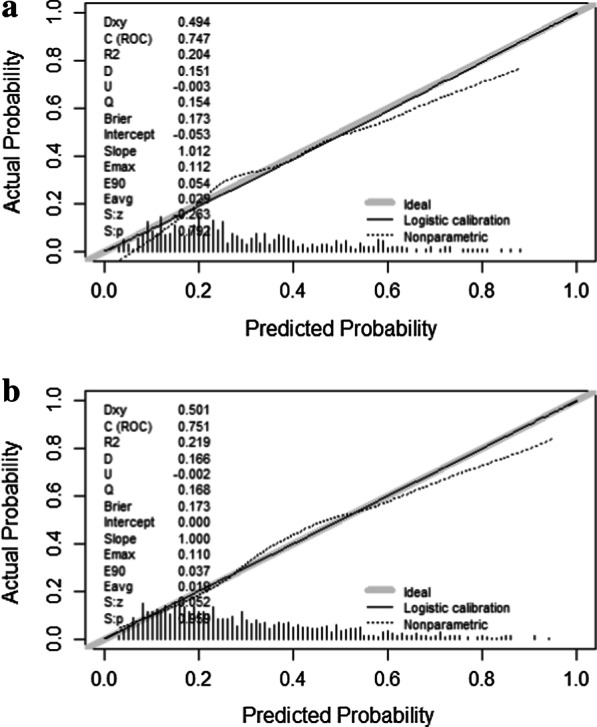
Calibration curves constructed by bootstrap approach in the training set (**a**) and validation set (**b**). In both sets, the apparent curve and bias-corrected curve slightly deviated from reference line, but a good conformity between observation and prediction is observed

Calibration refers to the agreement between observed outcomes and predictions [16], We adopt Hosmer–Lemeshow test and calibration plots to evaluate the calibration of the prediction model.Through Hosmer–Lemeshow test, χ^2^ of training set is 11.89 (*p* = 0.22) and χ^2^ of validation set is 12.13 (*p* = 0.21).Calibration curves of the training and validation sets are displayed in Fig. 4, with the bootstrap method used to form the curves after bias corrections. Conformity between predictions and observations in the calibration plot was satisfactory in both sets (Fig. 4), as the bias-corrected curve and apparent curve both just deviated slightly from the reference line.

### Clinical use of the nomogram

**Fig. 5:**
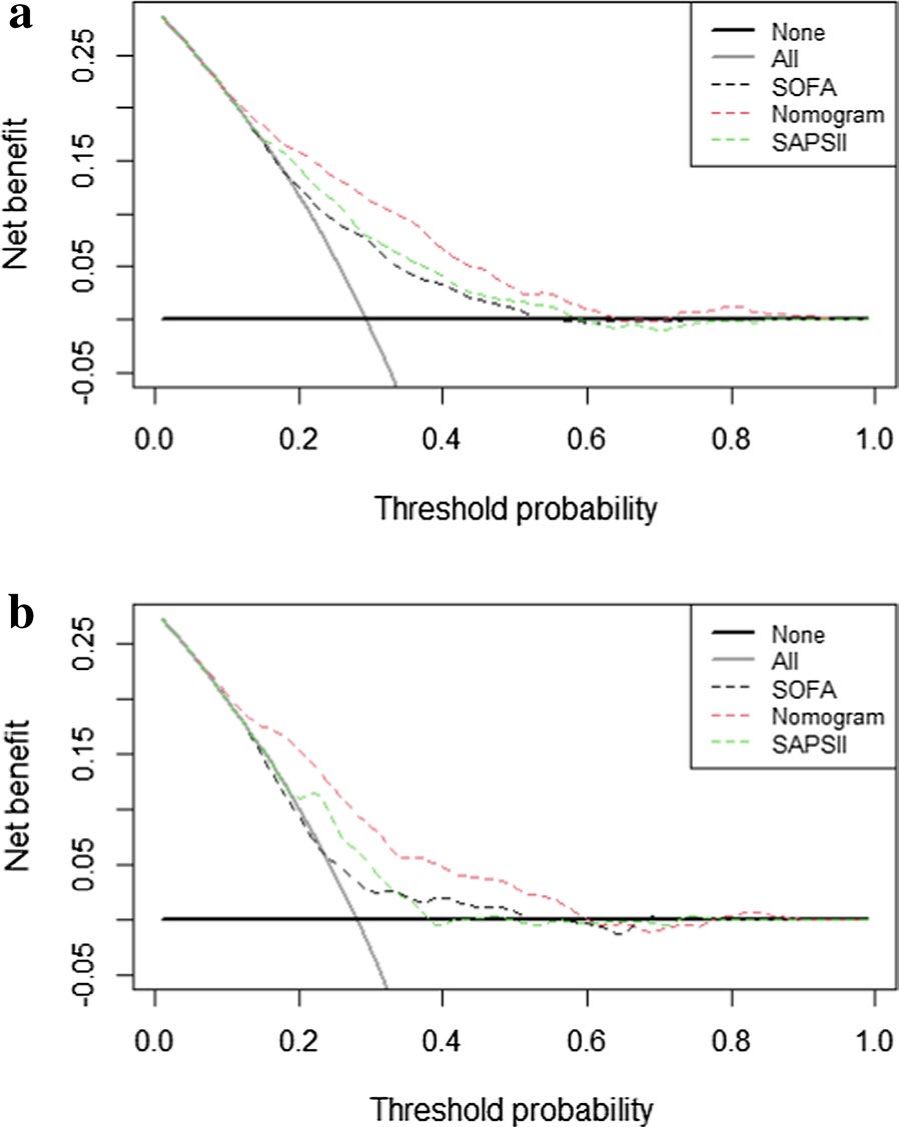
The DCA curve of medical intervention in patients with the nomogram, SOFA, and SAPSII in the training et (**a**) and validation set (**b**)

DCA was used to evaluate the clinical benefits of the nomogram, with SAPSII and SOFA score used as the reference. In both the training and validation sets, interventions based on the nomogram could provide better prognoses than the SOFA score and SAPSII when the probability threshold was between 0.1 and 0.6 (Fig. 5).

## Discussion

Since the introduction of a clinical definition for sepsis, physicians have been very concerned about its diagnosis and treatment due to its association with a high fatality rate, poor quality of life, and huge economic burden to patients [17]. Sepsis mainly induces systemic organ tissue damage from an inflammatory reaction, and capillary endothelial damage and loss of regulation of peripheral circulation are the key processes for sepsis developing into septic shock. Leukocyte oozing, coagulation dysfunction, and capillary dilation from inflammation are the main physiopathological factors associated with widespread tissue edema [18]. During sepsis development, collapse of pulmonary capillaries causes large amounts of protein-rich edema fluid to leak into the interstitial compartments of the lung [19]. The lung is therefore one of the most vulnerable organs during sepsis. Treating patients with sepsis who have lung infection is more difficult, and their mortality rate is higher [6, 7].

There has been recent interest in determining the factors related to the outcome of sepsis. Lactic acid, renal insufficiency, thrombocytopenia, lung infection, and high fever are considered risk factors for poor sepsis outcomes [6, 20–22], while plasma albumin and plasma IgG levels can be protective factors [23, 24]. However, there are relatively few studies on the risk factors related to the prognosis of sepsis patients with lung infection. The present multivariate logistic regression analyses of sepsis patients with lung infection from the MIMIC-III database indicated that the variables closely related to patient prognoses include vital signs (age, SAPSII, heart rate, mean BP, body temperature, and SpO2), test results (BUN, bicarbonate, creatinine, lactate, INR, TnT, NLR, and blood culture), complications (cancer, renal failure, liver disease, and organ transplantation), and intervention strategies (vasopressors, MV, and CRRT use). Among these variables, the OR values of body temperature, SpO2, and bicarbonate were less than 1, indicating that they are positively correlated with in-hospital survival of patients with sepsis and lung infection. Vincent and his colleagues found that the mean blood pressure was positively correlated with the survival rate of sepsis patients [25]. Such a correlation was also present in our study, but with an OR value of 1.07 (95% C.I.: 0.99–1.16), which may be due to the different sample populations.

Patients with sepsis have higher in-hospital mortality when lung infection is also present, and clinical treatments for them are more difficult. While current sepsis guidelines do not recommend detailed treatment methods and evaluation programs for patients with different infection sources and complications. There are some systems for clinically evaluating sepsis patients, such as SOFA score, qSOFA, SAPSII, APACHE III and APACHE IV, SOFA score and qSOFA are mostly used for early and rapid disease assessments of sepsis patients [10]. Compared with the SOFA score, SAPSII has improved discrimination, calibration, and predictive power for mortality in sepsis patients, which has been recommended for the identification and mortality prognosis of sepsis patients by Sepsis 3.0 [26]. The prediction efficacy of APACHE III and IV models for sepsis and septic shock patients performed unsatisfying according to Ajay Somabhai Dabhi’s study [27] and the efficacy of APACHE III, APACHE IV and SAPSII for predictions around sepsis patients with lung infection are uncertain. To meet the needs of clinical practice and to accurately understand sepsis development, some scholars have combined scoring systems and biomarkers to predict sepsis patient mortality. Seo et al. [28]. constructed a clinical predictive model for the 28-day mortality of sepsis and septic shock patients, with approximately 62% of the sepsis patients in the sample having lung infections. The variables in the model were hypoalbuminemia, low base excess values, and respiratory rate, and the model exhibited effective discrimination and calibration. However, those authors did not validate their predictive model, and so we extracted the clinical data of patients with sepsis and lung infection from the MIMIC-III database, applied logistic regression to determine the risk factors of in-hospital mortality, confirmed the prediction model and constructed a nomogram, and performed validity and calibration evaluations of the clinical model. Some studies have demonstrated the benefits of the latest method of DCA and recommend its use [29, 30], DCA is a method to evaluate prediction models by calculating the clinical net benefit. The results of our study showed that in both the training and validation sets, interventions based on the nomogram could provide better prognoses than the SOFA score and SAPSII when the probability threshold was between 0.1 and 0.6. We adopt Hosmer–Lemeshow test and calibration plots to evaluate the calibration of the prediction model [14, 16], the results of both test demonstrated our model’s calibration ability is satisfied.

Liver disease had the largest weighting factor in our model, indicating that it is the most significant predictor for the in-hospital mortality of patients with sepsis and lung infection. The morbidity of sepsis patients with liver disease is 30–50%, which is much higher than that of general patients with sepsis [31]. It is currently believed that the liver prevents sepsis from aggravating damage to tissues and organs mainly by removing bacteria and regulating inflammatory factor metabolism. Kupffer cells in the liver have an immune defense effect of removing bacteria and dissolving toxins [32]. Studies have indicated that liver damage may amplify lung inflammatory responses to bacterial infection coming from sepsis. Siore et al. used lipopolysaccharides to perfuse the lungs and livers of piglets, and found that when the liver and kidney were perfused simultaneously, nitric oxide, tumor necrosis factor alpha, and interleukin-6 levels are elevated in the lung, causing pulmonary edema [33]. When proinflammatory cytokines are synthesized during sepsis, the liver also secretes anti-inflammatory cytokines such as interleukin-10, transforming growth factor β, and glucocorticoids concomitantly, which may prevent continuous organ injury associated with the proinflammatory cytokines, but may cause severe immunosuppression. Infection immunity and endotoxin clearance insufficiency have been observed in patients with sepsis, acute liver failure, and cirrhosis [34].

Our study found that cancer and the NLR are closely related to patient mortality, and these factors are closely related to the immune status of each patient. Many studies have indicated that the incidence and mortality rates of sepsis in immunocompromised patients are much higher than in healthy subjects [35, 36]. Through the immune checkpoint pathway and the secretion of immunosuppressive factors, cancer can suppress the innate immunity and adaptive immunity of the host [37, 38]. Ni et al. suggested that the NLR was related to sepsis patient mortality [39], but their research results indicate that NLR has a negative correlation with sepsis patient mortality, contrary to our results. This may be because the study population and selected variables differed between the studies.

Compared with the SOFA score and SAPSII, our clinical model has better prediction and discrimination performance, and the verification performed through IDI, NRI, Hosmer–Lemeshow test, calibration plots, and DCA demonstrated that our model has good discrimination, calibration, and validation for predicting in-hospital mortality in target patients.

Our study had some limitations. First, since different methods are used to diagnose sepsis, such as the Martin criteria, it is necessary to further verify the efficacy of our model based on these criteria. Second, our nomogram was obtained through retrospective observation research from MIMIC-III database and according to our inclusion criteria some population are excluded from our study, this may limit the generalizability of our model (such for patients elder than 80 and patients in emergency department), and including additional factors in the model may affect the prediction results(for example, the time of receiving antibiotics). Finally, we only conducted an internal validation by this database, external validation based on our own data should be performed in the future study to further validate the robustness and performance of the nomogram.

## Conclusion

The present novel nomogram that includes the variables of age, lactate, temperature, oxygenation index, BUN, GCS score, liver disease, cancer, organ transplantation, TnT, NLR, and CRRT, MV, and vasopressor use can be applied to accurately predict the in-hospital mortality of ICU patients with sepsis and lung infection. Treatment strategies aimed at improving the factors considered relevant in the model can improve in-hospital survival rates for these ICU patients.

## Supplementary Information

**Additional file 1.** TRIPOD checklist is provided for the prediction model development and validation.

## Data Availability

All data generated or analysed during this study are included in this published article.

## Abbreviations

MIMIC: Medical Information Mart for Intensive Care
SOFA: Sequential Organ Failure Assessment
SAPSII: Simplified Acute Physiology Score
CHF: Congestive heart failure
COPD: Chronic obstructive pulmonary disease
MBP: Mean blood pressure
NLR: Neutrophils to lymphocytes ratio
TnT: Troponin T
MV: Mechanical ventilation
CRRT: Continuous renal replacement treatment
CVP: Central vein pressure
AUROC: Area under the receiver operating characteristic curve
IDI: Integrated discrimination improvement
DCA: Decision curve analysis
NRI: Net reclassification index
